# Odor mixture training enhances dogs' olfactory detection of Home-Made Explosive precursors

**DOI:** 10.1016/j.heliyon.2018.e00947

**Published:** 2018-12-08

**Authors:** Nathaniel J. Hall, Clive D.L. Wynne

**Affiliations:** a Texas Tech University, USA; b Arizona State University, USA

**Keywords:** Neuroscience, Veterinary science

## Abstract

Complex odor mixtures have traditionally been thought to be perceived configurally, implying that there is little identification of the individual components in the mixture. Prior research has suggested that the chemical and or perceptual similarity of components in a mixture may influence whether they can be detected individually; however, how experience and training influence the ability to identify individual components in complex mixtures (a figure-background segregation) is less clear. Figure-background segregation is a critical task for dogs tasked with discriminating between Home Made Explosives and very similar, but innocuous, complex odor mixtures. In a cross-over experimental design, we evaluated the effect of two training procedures on dogs' ability to identify the presence of a critical oxidizer in complex odor mixtures. In the Mixture training procedure, dogs received odor mixtures that varied from trial to trial with and without an oxidizer. In the more typical procedure for canine detection training, dogs were presented with the pure oxidizer only, and had to discriminate this from decoy mixtures (target-only training). Mixture training led to above chance discrimination of the oxidizer from variable backgrounds and dogs were able to readily generalize performance, with no decrement, to mixtures containing novel odorants. Target-only training, however, led to a precipitous drop in hit rate when the oxidizer was presented in a mixture background containing either familiar and/or novel odorants. Furthermore, by giving Target-only trained dogs Mixture training, they learned to identify the oxidizer in mixtures. Together, these results demonstrate that training method has significant impacts on the perception of components in odor mixtures and highlights the importance of olfactory learning for the effective detection of Home Made Explosives by dogs.

## 1 Introduction

Olfactory figure-background segregation is the identification of a target odorant against a complex odor background. In natural environments, animals need to be able to identify target odorants, such as food items, even against complex and variable backgrounds [1, 2, 3, 4, 5]. In nature, target and background odor plumes can be temporally and or spatially segregated thereby simplifying olfactory figure-background segregation [1, 2, 5, 6]. A more complex task, however, occurs when a single component needs to be distinguished from a mixture of simultaneously presented odorants [7, 8]. Some researchers have questioned whether identifying the components of such a mixture is possible at all [8]. Mixtures tend to be perceived configurally, such that the mixture produces a unique percept distinct from the constituent elements, and this may vary depending on the chemical similarities of the components in the mixture [9, 10, 11, 12, 13, 14].

The ability to elementally segregate complex odor mixtures is critical for explosives detection dogs, which are required to detect explosives when buried, concealed, and/or covered with masking odorants [15, 16, 17]. Figure-background segregation is even more critical for dogs' detection of improvised or homemade explosives (HMEs). HMEs can be composed of a nearly unlimited range of components producing complex odor mixtures. For example, Ammonium Nitrate (NH_4_ NO_3_; AN) can be combined with a wide range of organic compounds as fuels such as fuel oil, icing sugar, and other organic materials. This makes the olfactory target a highly variable odor mixture.

Unfortunately, the identification of individual components in mixtures, at least for humans, can be quite challenging [7, 8]. Furthermore, it is not clear how a variety of factors may combine to determine whether odor mixtures are perceived as individual elements (elementally), or configurally [12, 13, 14, 15].

Rokni and colleagues [3], recently demonstrated that mice can identify the presence of a target odorant in up to 14 component mixtures when the components are presented simultaneously. Rokni et al. trained the mice using highly variable backgrounds that changed from trial to trial. This rich odor mixture training is likely important for the mice to perform this figure-background segregation, however, this was not explicitly tested in Rokni et al., because they did not include a group receiving a different form of training as a comparison. Furthermore, it is not clear whether performance would generalize to mixtures containing new components that were not used during training. The findings of Rokni and colleagues [3] importantly demonstrate the possibility of figure-background segregation in rodents. Perhaps similar processes extend to olfactory processes in dogs. This would have important implications for detection dogs and suggest the ability to perform an elemental separation likely depends on training history [3, 15, 18, 19].

Together, these results suggest that for dogs to show optimal performance in detecting an odor target in highly variable and complex backgrounds, they need to be trained with a variety of odor mixtures with and without the target. This is in contrast to a Target only training procedure in which dogs are trained to detect the primary oxidizer (the Element/Target) and to not respond to ‘distractors’ or odors without the target. Some research suggests that these Target-only based procedures may not be optimal. For example, dogs trained to pure potassium chlorate did not generalize to potassium chlorate based explosives and mixtures [20]. Furthermore, dogs trained to AN, did not readily generalize to chemically related salts or even different grades of AN such as fertilizer grade [21].

The ability to identify a critical oxidizer (such as AN) in a complex odor mixture is likely influenced by prior experience and training history, but no direct tests have been done in this critical context [3, 15, 18, 19, 20]. The aim of this study is to evaluate the effect of form of training on configural olfactory processing and detection of an oxidizer target in odor mixtures. Dogs were trained using an automated olfactometer in a go/no-go procedure using a configural or “Mixture-training” procedure or an Element or “Target-only” training procedure. We compared dogs' subsequent detection of the oxidizer in mixtures with familiar and unfamiliar components.

## 2 Methods

### 2.1 Subjects

Six mixed breed dogs participated in the present study. Dogs were between 8 months and 3 years of age at the start of the experiments. Two of the dogs showed poor motivation for food during training and were subsequently excluded from testing. The remaining subjects were four mixed breed dogs (three females and one male) between 13.5 and 23 kgs. Dogs' backgrounds were unknown but all dogs were presumably naïve to detection training and adopted and housed in a university training facility for the purposes of the study. Dogs received twice daily walks, social enrichment, and training and were adopted at the end of the study. Procedures were reviewed and approved by the Arizona State University Institutional Animal Care and Use Committee.

### 2.2 Equipment

**Fig. 1:**
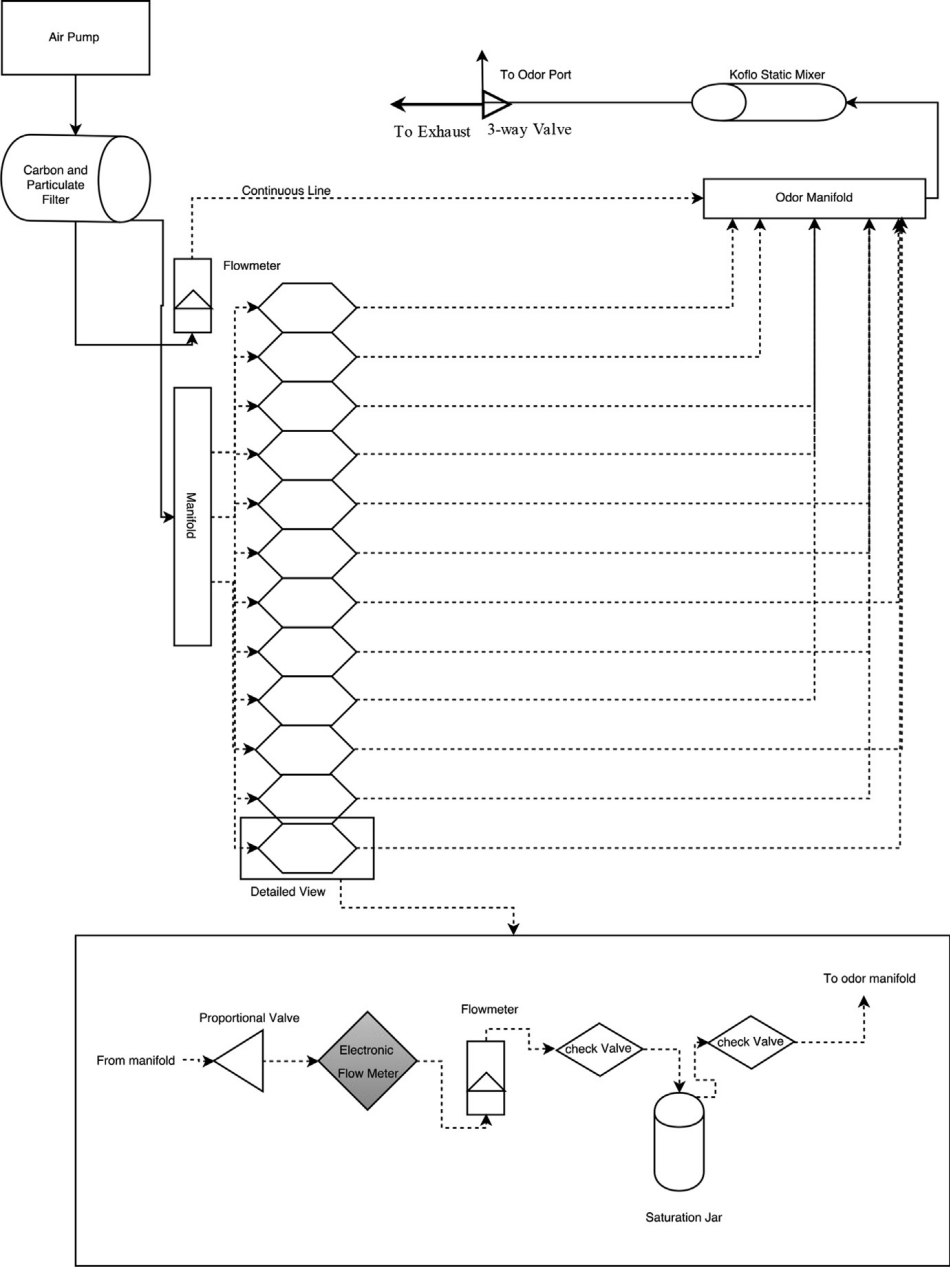
Diagram of the olfactometer design. Shows the trace of the airflow through the olfactometer system. Each channel is measured and controlled via a mass air flow meter and a proportional valve.

Dogs were trained to use an automated 12 channel dynamic-dilution computer-controlled olfactometer (Fig. 1). The olfactometer controlled presentation of odor mixtures to dogs with proportional valves and electronic mass air flow meters. Each channel held a specific odorant in a saturation jar. The odorant was presented by passing clean air through the jar, forcing the odorant headspace into a mixing manifold. Each channel was regulated independently using a feedback control mechanism between the mass air flow controller and proportional valve. The feedback control required a maximum of 3 s to obtain accurate air flows for all channels. During this settling time, an upstream valve insured all odorants were ported to the exhaust system. Only when the system was ready with accurate dilutions, was the odor presented to the odor port where the dog could sniff it. Some *a priori* restrictions were placed on odor generation and are described in the odor mixtures section below. The odor mixture was always diluted with a final clean air continuous line, so that the odorant comprised nominally 33% of the final presentation.

**Fig. 2:**
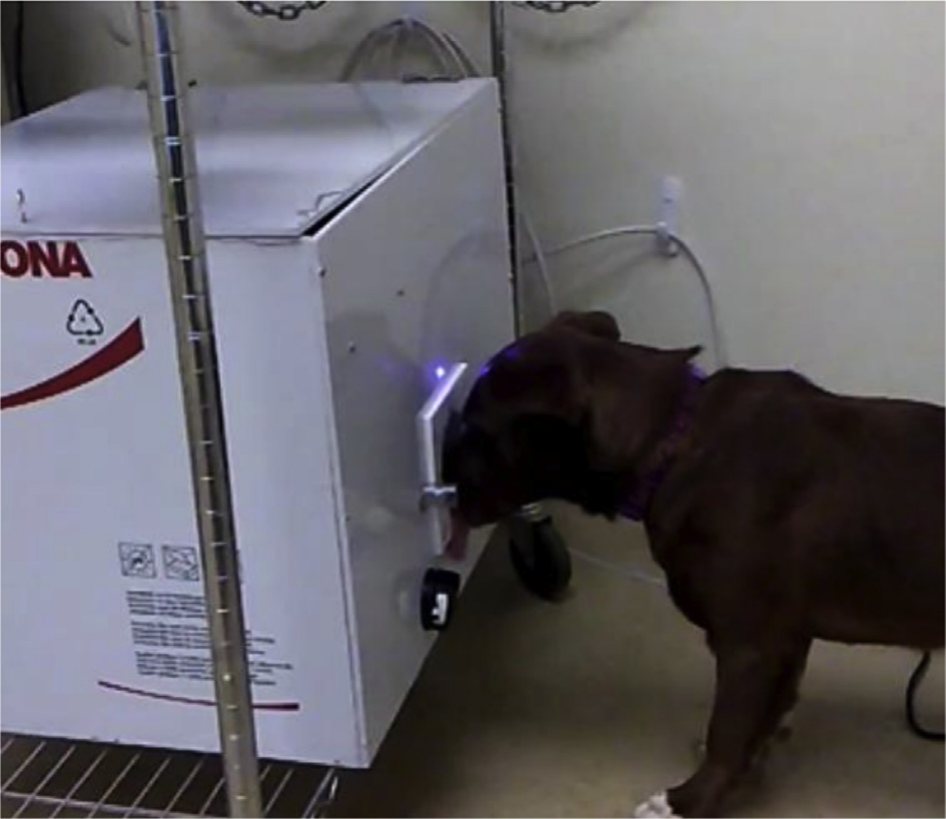
Dog working on the olfactometer. The dog has its nose in the odor port to initiate a trial.

All components that came in contact with odorants, except the check valves, were comprised of Teflon (PTFE), glass, or stainless steel. Check valves were changed for each odorant to prevent cross-contamination. Fig. 2 shows a dog interacting with the olfactometer. At the front was a Teflon (PTFE) odor port where the odor was presented. Infrared (IR) sensors detected nose entries into the port. An LED above the port illuminated whenever the olfactometer was active for a nose poke. The LED was off during the inter-trial intervals. Below the odor port was a plastic tube which delivered food reinforcers for correct responses. At the back of the olfactometer (not shown), an exhaust tube evacuated odorants from the odor port to a standard laboratory fume hood.

To reduce contamination between trials, there was always a 15s inter-trial interval. During this interval, an upstream valve to a clean air line was pulsed repeatedly every 20 ms for 15 s to help clear the lines. At the end of every day, all common odor lines and components were washed in hot water, rinsed with ethanol, and placed in an oven overnight between 85 to 100**°**C.

### 2.3 Odorants

For all odorants, their manufacturer and purity are shown in Table 1. All odorants except AN and H_2_O_2_ were diluted to 0.01% v/v in mineral oil. AN was not diluted and 10 g was placed in the saturation jar. AN was not diluted because of its low vapor pressure [22] and reduced concentration of this odorant in comparison to all others. H_2_O_2_was diluted in deionized water to 10% v/v to reduce the dogs' exposure to H_2_O_2_. A total of 10 ml of the odorant dilution was placed in the saturation jars. Odorant dilutions were prepared and replaced weekly throughout all testing.

**Table 1:** Identity and manufacturer of odorants; including phases of the experiment where used.

Name	Purity	Manufacturer	Phase Used
Ethyl Propionate	99%	Sigma-Aldrich	AN Training Distractor
Pentyl Acetate	99%	Sigma-Aldrich	AN Training Distractor
1-Hexanol	98%	Sigma-Aldrich	AN Training Distractor
1-Pentanol	99%	Sigma-Aldrich	AN Training Distractor
4-Allylanisole	98%	Sigma-Aldrich	AN Training Distractor
(R)-(+)-Limonene	97%	Sigma-Aldrich	AN Generalization Distractor
(S)-(-)-Limonene	96%	Sigma-Aldrich	AN Generalization Distractor
Allyl butyrate	98%	Sigma-Aldrich	AN Generalization Distractor
Hexyl Tiglate	97%	Sigma-Aldrich	AN Generalization Distractor
Ethyl Tiglate	98%	Sigma-Aldrich	AN Generalization Distractor
1-Butanol	99.8%	Sigma-Aldrich	H2O2 Training Distractor
1-Propanol	99.7%	Sigma-Aldrich	H2O2 Training Distractor
Ethyl Valerate	98%	Sigma-Aldrich	H2O2 Training Distractor
Isobutyl Propionate	98%	Sigma-Aldrich	H2O2 Training Distractor
Isopropyl Tiglate	98%	Sigma-Aldrich	H2O2 Training Distractor
(S)-(+)-Carvone	96%	Sigma-Aldrich	H2O2 Generalization Distractor
Methyl salicylate	99%	Sigma-Aldrich	H2O2 Generalization Distractor
2-Ethylhexanal	97%	Alfa Aesar	H2O2 Generalization Distractor
2-Phenylethanol	98%	Alfa Aesar	H2O2 Generalization Distractor
(R)-(-)-Carvone	98%	Alfa Aesar	H2O2 Generalization Distractor
Ammonium Nitrate	99%	Sigma-Aldrich	Target 1
Hydrogen Peroxide	30%	Sigma-Aldrich	Target 2

### 2.4 Odorant mixtures

A variety of odorant mixtures was dynamically created by mixing odor head spaces. The odor mixtures were generated using the following procedure. First, the number of components in the mixture was chosen from a range from 1 to 5, with a probability of one component set at 25% and the probability of all other numbers of components set uniformly at 18.75%. Next, the trial was determined to either contain the target or not depending on whether the target was being mixed or not in the respective phase of the experiment. All other odorants of the mixture were then selected from the odorants available with equal probability (6 odorants were available during training and an additional 5 novel odorants were available during generalization testing). Next, the concentration for each odorant was selected. The flow rate of the odor line was restricted to a total of 1 L/min and each individual line was permitted to provide between 0.2 to 0.9 L/min of the 1 L/min total. The odor flow rates were randomly allocated with the restriction that they must sum to 1 L/min or less. If the flow rates summed to less than 1 L/min, then the remaining flow rate was allocated to a clean air channel so that the final air flow from the odor line was always 1 L/min. This line met with the continuous line (always 2 L/min) providing a final airflow of 3 L/min to the odor port. Concentrations and odor identity were determined in this manner to provide dogs with a wide range of variability in both odor type and concentration which might be expected in a Home-Made Explosive.

### 2.5 Training

Dogs were initially trained to insert their nose into the odor port breaking the IR beam. Dogs were shaped using an automated program to hold their nose in the odor port for 7 s before receiving a treat. Once dogs were successful in holding their nose in the port, discrimination training began. Dogs were required to initiate a trial by placing their nose in the odor port. The olfactometer then began to make the correct odor mixture which took 3 s. All odorant during this time was ported to the exhaust, and therefore dogs were not receiving any odor at this point. After 3 s, the final upstream valve directed the odorant towards the dog. The dogs were then required to maintain their nose in the port for 0.5 s. If the dog did not maintain its head in the odor port for 0.5 s, the trial was cancelled. If dogs maintained their nose in the port, breaking the IR beam for at least 0.5 s, a go/no go trial was initiated. If a target odorant was present, dogs were required to maintain their head in the odor port for another 3.5 s. If the odor was not present, dogs were required to remove their head for at least 3.5 s. If dogs made a correct response (a ‘hit’ or ‘correct rejection’) they received a piece of food from the automated feeder. Otherwise, no food was presented and the 15 s inter-trial interval began. Across all phases of the study, the ratio of target to non-target trials was held constant at .5.

Data were collected via a desktop computer connected to the olfactometer via USB. Dogs were initially trained to respond to vanilla extract diluted to 1% v/v in distilled water. Once they reached 75% accuracy or higher, the dilution was decreased gradually to 0.01% v/v. On attaining 75% accuracy on this dilution, dogs were transitioned to AN as the target odorant.

### 2.6 Procedure

We utilized a cross-over experimental design to evaluate whether mixture training or training to pure targets (‘Target-only training’) led to the greatest generalization to novel odor mixtures. The four dogs were randomly assigned to two groups, A and B (see Table 2). Both groups were first trained and tested using AN as a target. After testing with AN, the training assignments were crossed between groups and all dogs were trained with H_2_O_2_ as the target. Training and assessment procedures were identical for both target odorants. Throughout training and testing phases dogs received two 40 trial sessions per day (one in the morning and one in the afternoon).

**Table 2:** Experimental design. Dogs that started in Target-only training switched to Mixture training, whereas dogs that started in Mixture training switched to Target-only training.

	Target-only Training	Mixture Training
Ammonium Nitrate	Group A	Group B
Hydrogen Peroxide	Group B	Group A

All dogs were first trained to simply detect the target odorant (50% dilution, 16.5% overall when mixed with the continuous line) at above chance levels (at least 50 out of 80 correct trials per day per binomial test) for six consecutive days. The probability of a target vs non-target odorant was 50%. During these Baseline sessions, dogs were required to alert to the pure target odorant and reject clean air as the distractor. Once dogs showed a stable performance for at least six days, they were changed to experimental training for 25 days. The dogs in the Target-only training group were trained to the pure target (at 50% dilution) and to ignore a variety of distractors as non-targets. Distractors were generated using five different pure odorants from Table 1 and the mixture algorithm described in detail above. Dogs in the Mixture group were trained with odor mixtures with the target and with odor mixtures of non-target stimuli. These odor mixtures were created from the target odorant and five distractors using the odor mixture algorithm noted above. Target trials and non-target stimuli were identical kinds of odor mixtures differing only in whether the mixture included the oxidizer odor or not.

Following 25 days of training, all dogs underwent testing for generalization. The goal of these trials was to test whether dogs that were target-only trained would generalize to mixture trials at similar rates to mixture trained dogs, and to compare how both groups of dogs generalized to target odors that contained novel components they had never experienced during training. Thus, during generalization testing, all groups were transitioned to Mixture training. In addition, 25% of the trials were probe trials in which from one to all of the distractor odors in the mixture was an odorant from Table 1 that had never previously been used in training. This assessment was conducted to more closely replicate an applied scenario in which dogs trained using a ‘target-only’ method might be expected to detect mixtures with previously familiar components (which reflect mixture training trials) or may include novel components (probe trials).

Probe trials were non-differentially reinforced (food was given whether responses were correct or not) to evaluate spontaneous responding. All probe responses were reinforced to maintain the reinforcement rate and sustain motivation and responding during generalization tests which we anticipated would show lower accuracy rates. Each dog received six days of generalization training.

After all dogs completed training and testing with AN, they changed to the alternative condition and were trained under baseline conditions to H_2_O_2_ such that dogs learned to respond to H_2_O_2_ compared to clean air at above chance levels for six consecutive sessions. Once reaching this criterion, the respective procedures were repeated with the opposite assignment of groups completing the crossover design (Table 2).

### 2.7 Control

At the end of each testing phase for both AN and H_2_O_2_, a day of control testing was conducted. During control testing, all the odorants were cleaned from the olfactometer, and the dogs were required to complete two sessions with no odorants presented. The same olfactometer channel and jars that held the target odorant during testing trials was assigned as the target for control trials, although no odorant was intentionally placed. Thus, the olfactometer arrangement was identical to the testing procedures, except that no odorants were presented. This control tested for the possibility that dogs were utilizing extraneous cues during testing such as discriminating between subtle ‘clicks’ of valves opening or other unintentional non-olfactory cues.

### 2.8 Statistical analysis

Logistic mixed effect models using R [16] and the *lmerTest* [17] and *lsmeans* [23] packages were run. For all models, subject ID was included as a random intercept. The lsmeans package was utilized to conduct Tukey corrected post-hoc tests. To compare overall accuracy differences when transitioning from baseline (target vs. blank) to respective training conditions, we fit a logistic mixed effect model, in which whether a response was correct/incorrect was predicted by the oxidizer type (AN or H_2_0_2_), the trial type (baseline or training), the group (mixture or target only training) and the interaction between trial type and group (See raw data: “baseline_raw_data.csv”). To test for differences in the hit rate or false alarm rate, identical predictors were fit to models in which the response variable was whether the dog alerted for trials where the odorant was present (hit rate) or the odorants was absent (false alarm rate).

To compare the effect of training group on performance during training and generalization, we focused analyses on the last five days of training and the first day of generalization testing (∼4,000) trials (see raw data: “generalization_raw_data.csv”). A logistic mixed effect models was fit in which the response (correct/incorrect) was predicted by the oxidizer type (AN or H_2_0_2_), the trial type (training, generalization mixture trial, probe trial), the group (mixture or target only training) and the interaction between trial type and group. Identical models for hit rate and false alarm rate were fit with similar parameterization but focused on whether a dog alerted (yes/no) for trials in which the odorant was present (hit rate) or absent (false alarm rate).

## 3 Results and discussion

**Fig. 3:**
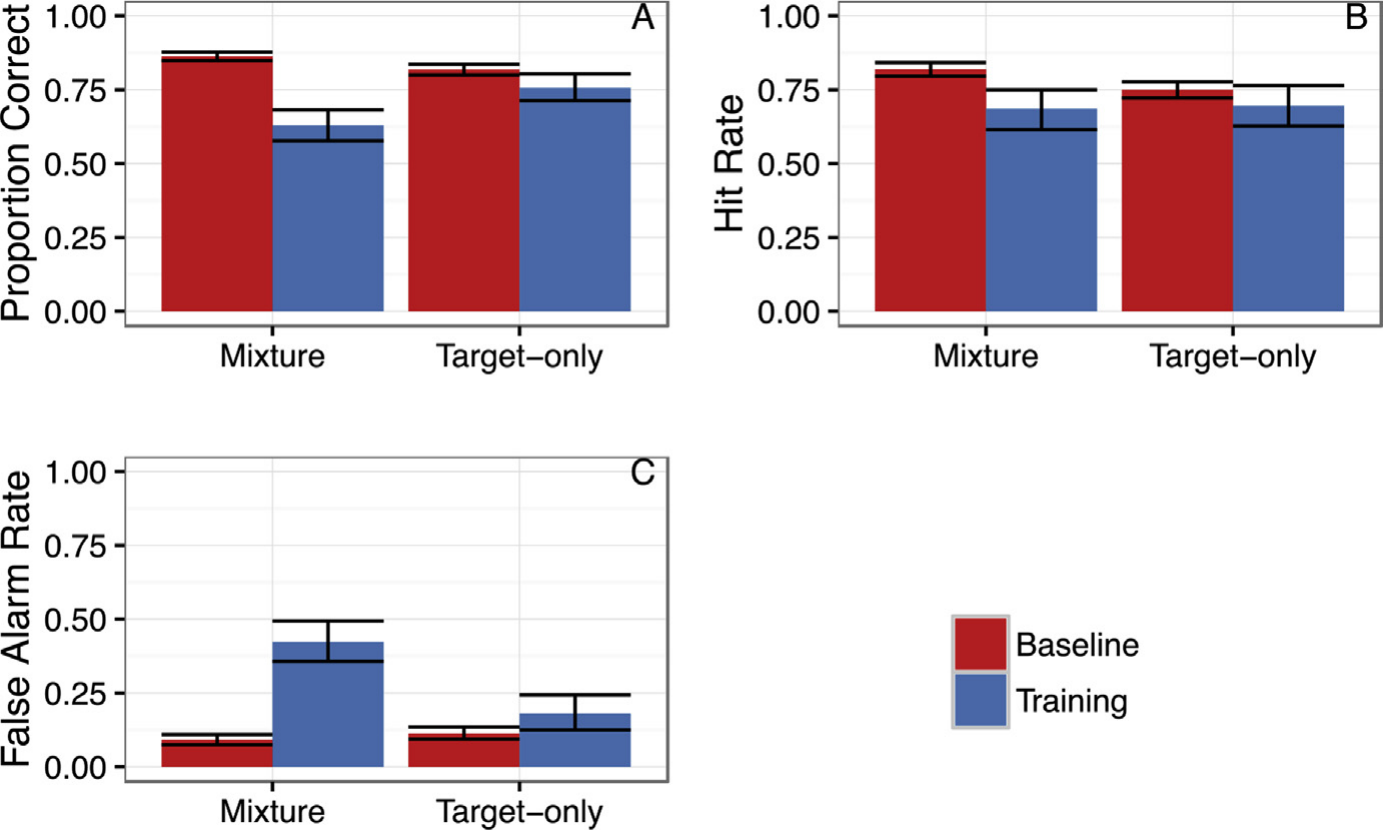
Transition from Baseline to Training. Figure shows the change in performance when the experimental training was implemented with 95% confidence intervals (boot-strapped) for overall proportion correct, hit rate and false alarm rate. Bars show the mean for all Baseline trials (target odor vs. blank) and all training trials for the Mixture and Target-only trained groups.

Mixture training proved to be overall more difficult to learn than the Target-only training. There was a larger drop in initial performance when Mixture training was introduced to the dogs compared to when Target training was introduced from baseline indicated by a significant group (Target vs Mixture) by trial type (baseline vs. training) interaction (*z* = 5.00, *p*< .001; Fig. 3A). This larger drop in accuracy for the Mixture training group was due to a disproportionate increase in the false alarm rate (incorrect alerts in the absence of the target) when odor mixtures were introduced as targets as a group by trial type interaction was significant for the false alarm rate (Fig. 3B-C; z = 4.69, *p* < .001) but not the hit rate (z = 1.77, *p* = .07).

**Fig. 4:**
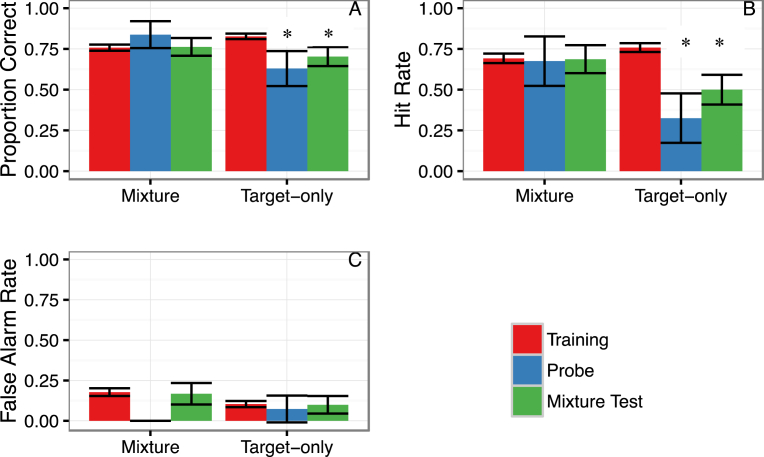
Generalization from the last five days of training (“Training”) to the first generalization session for dogs in the respective training groups (Mixture training or Target-only training). Trials from the generalization testing session shows performance when dogs were presented with familiar odor mixtures (“Mixture Test”) and probe trials in which odor components were novel to the dogs (“Probe”). Bars show the mean and error bars show the 95% CI from boot strapped estimates. A: shows the group and trial type effect on overall accuracy (proportion correct). B: shows the same effects on hit rate (accuracy when target present) and C: shows the same effects on false alarms (incorrect indications when target was absent). * indicates a significant difference (p < .05) from Training trials.

Although Mixture training proved more difficult to learn initially, we evaluated whether it led to enhanced detection of the oxidizer in variable mixtures. Analyzing the performance during the last five days of training and the first day of generalization testing (∼4,000 trials in total), we found a significant interaction between the group (mixture vs target only trained) and the trial type (training, mixture test and probe trials) by comparing a model with and without an interaction term (χ^2^ = 26.98, df = 2, *p* < .001). Least Square Means Post-hoc tests indicated that dogs trained to the target only had a significant drop in performance when required to detect the oxidizer in the presence of either familiar (*z* = 4.46, *p* < .0001) or novel odor components (*z* = 4.63, *p* < .001). In contrast, Mixture trained dogs showed no such drop with either the familiar (*z* = −0.18, *p* = .999) or novel odor mixtures (z = −1.67, *p* = .55; See Fig. 4A). Although the Target-only trained group outperformed the Mixture-trained group during training (z = 5.29, *p* < .0001), the Mixture trained group outperformed the Target only group on novel probe trials (*z* = 3.00, *p* = .032), and there was no significant difference in Mixture test trials (*z* = −1.47, *p* = .68).

Critically, the decrease in performance for the Target-only group was largely due to a drop in the hit rate (detection when the target is present, see Fig. 4B). Although the Target-only group showed a higher hit rate during training (*z* = 3.94, *p* = .001), the Mixture group showed a higher hit rate at test on both novel probe trials (*z* = 3.04, *p* = .028) and on mixture test trials (*z* = −3.02, *p* = .030). Thus, the Target-only training led to a *failure* to recognize the target in odor mixtures. In contrast, dogs that received the Mixture training were able to recognize the target odor when it was embedded in either familiar (*z =* 0.11, *p* = .99) or novel odor mixtures (i.e., successful figure-background segregation; *z* = 0.71, *p* = .98) at rates equal to those found during training, and at higher rates than those produced by Target-only training. The false alarm rate remained unchanged in both groups, suggesting that the method of training largely influenced detection of the oxidizer's presence (hit rate) rather than its absence (false alarm rate; Fig. 4C).

### 3.1 Learning during generalization test

**Fig. 5:**
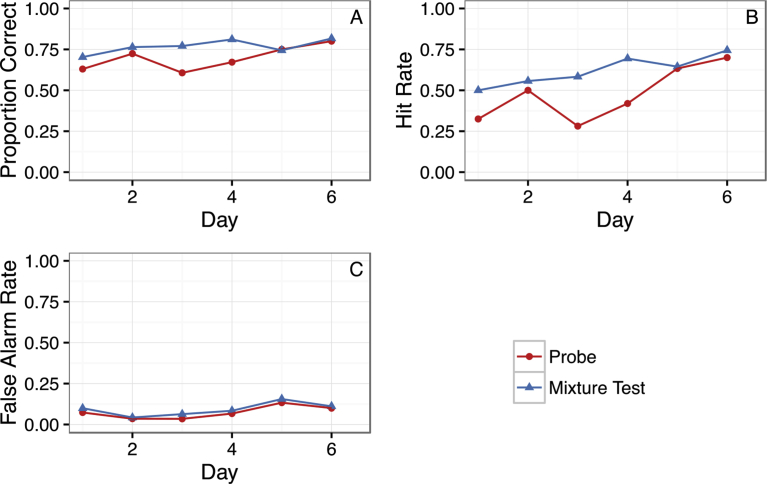
Learning during generalization testing. Figures show changes in accuracy (A), hit rate (B) and false alarm rate (C) across the generalization testing days for the Target-only trained group.

To further confirm that successful figure-background segregation is related to the training method, we evaluated whether the Target-only trained group learned the figure-background segregation across the reinforced mixture trials presented during the generalization sessions. We fit a logistic mixed effect model for the accuracy (correct/incorrect) for the target-only group predicted by the odor type and day of generalization testing. Dogs showed significant improvement across the six days of testing (Fig. 5A, *z* = 2.02, *p* = 0.044), which is particularly evident in the increases in the hit rate across test days (Fig. 5B). Performance did not differ, however, between the mixture test and probe trials across all six generalization days although there was a trend for higher performance with more familiar components (*z* = 1.81, *p* = .07). This further confirms the important relationship between the specific training and the ability to perform a figure-background segregation of a complex odor mixture.

### 3.2 Control tests

To confirm that the present results were due specifically to the olfactory stimuli, following the last test session for each oxidizer, dogs were given a control test in which the odorants were cleaned from the olfactometer. Dogs were then tested under normal operation– just absent any intended odors. Dogs showed a precipitous drop in performance, with a mean accuracy of 45% during control sessions, indicating that the olfactory stimuli had been controlling performance.

These results indicate two important findings. First, the training method influences whether a target odorant in a complex odor mixture is unrecognizable (configural perception) or detectable (figure-background segregation). Second, dogs can perform the required figure-background segregation to differentiate potential HMEs from innocuous complex odor mixtures. This finding has critical significance for the detection of HMEs. Dogs trained to alert to only one component of an odor mixture will fail to recognize the oxidizer in an explosive mixture. Importantly, if the dog is trained with mixtures containing the oxidizer and similar mixtures not containing the target, dogs can generalize detection of the oxidizer even when mixed in novel odor mixtures. This suggests that training dogs to a critical component of HMEs needs to be done using a mixture-training procedure and not direct training to an oxidizer such as AN.

Recent research has demonstrated that mice can identify a target odorant in mixtures of up to 14 simultaneously presented components [3]. These authors trained the mice using variable backgrounds that changed from trial to trial. The results of the present experiment confirm that it is the type of training that is critical for successful figure-background segregation. By comparing a training procedure using odor mixtures to training with an equivalent number of trials to just the target, we demonstrate the importance of the training paradigm on the type of perceptual processing that's observed.

There are several important limitations to the present study worth considering. First, the overall sample size is small. Due to the extensive training required for these dogs to successfully respond to AN and H202 in mixtures under controlled laboratory conditions, only a few animals could be tested. Before generalized recommendations can be made for detection training programs, this study will need replication in a larger sample.

A further limitation is that when transitioning from training to the generalization tests, Target-only trained dogs were introduced to two new trial types (Mixture Test and probe trials) whereas mixture trained dogs were already familiar with Mixture Test trials from training. This may have led to a more extreme disruption in performance for target-only trained dogs. This was done, however, to mimic what might occur if a Target-only trained dogs is deployed and expected to detect any variety of explosives with familiar and unfamiliar components.

In addition, our probe trials were conducted such that any response was reinforced. This was done to maintain a higher reward rate during the generalization testing days, although probe trials are more commonly run under extinction conditions. It is unlikely this factor significantly contributed to the results given that false alarm rates remained low and that this difference was consistent across experimental conditions.

Overall, although further replication is needed across a larger sample, the present results indicate that dogs will not spontaneously recognize a trained component in an odor mixture after target only training. If recognition of a component in a mixture, however, becomes relevant to reward, then dogs will learn this discrimination. Such scenarios might arise in nature when one component in an odor mixture might signal the difference between something edible or toxic. Together, this highlights the importance of experience and learning on how complex olfactory stimuli are processed.

## Declarations

### Author contribution statement

Nathaniel J. Hall, Clive D.L. Wynne: Conceived and designed the experiments; Performed the experiments; Analyzed and interpreted the data; Contributed reagents, materials, analysis tools or data; Wrote the paper.

### Funding statement

This work was supported in part by a grant from the Office of Naval Research (N00014-15-1-2347).

### Competing interest statement

The authors declare no conflict of interest.

### Additional information

No additional information is available for this paper.

## Appendix A Supplementary data

baseline_raw_data baseline_raw_data

generalization_raw_data generalization_raw_data
